# The role of automated image co-registration and internal dosimetry in personalized medicine

**DOI:** 10.1590/0100-3984.2023.56.3e4-en

**Published:** 2023

**Authors:** Leonardo Alexandre-Santos, Lauro Wichert-Ana

**Affiliations:** 1 Section of Nuclear Medicine and PET/CT, Ribeirão Preto Medical School, University of São Paulo, Ribeiráo Preto, SP, Brazil. Email: lwichert@fmrp.usp.br.

Treatment with radioisotopes, also known as targeted radionuclide therapy (TRT), is challenging to implement safely and optimally in clinical practice^(1)^. It is based on administering a radioisotope that emits heavy (alpha and beta) particles. After administration, the radioisotope is deposited in the target organ through natural tropism or through transport by a vector molecule^(2)^. Cellular damage can occur as a result of the radiation emitted. The desired conditions to maximize exposure of the target organ and preserve adjacent tissues of varying radiation sensitivities are intimately associated with several factors that can be identified in advance during an internal dosimetry-based planning process.

The study “Validation of automated image co-registration integrated into in-house software for voxel-based internal dosimetry on single-photon emission computed tomography images”, conducted by Leitão et al.^(3)^ and published in the current issue of Radiologia Brasileira, sought to explore one of those factors and its impact on the definition of the estimated absorbed dose after nuclear medicine imaging, addressing image co-registration as an essential preprocessing step for effective planning of TRT. Commercial applications such as the Organ Level INternal Dose Assessment for EXponential Modeling (OLINDA/EXM) quantification platform offer end-to-end features for estimating the absorbed dose, promising to guarantee that the processes for estimating internal dosimetry factors will be more precise, rapid, and reproducible^(4)^. There is a growing demand for technologies that enable image co-registration for internal dosimetry calculations. Various studies have investigated the impact that the stages of image preprocessing intended for dose estimation, including spatial transformation of the original data, have on the patient^(5,6)^.

The Leitão et al.^(3)^ study introduces a technological advance by developing and testing an automatic co-registration method for single-photon emission computed tomography (SPECT) images, which could be integrated with their in-house software, designated NMDose-coreg. The authors investigated the need for fiducial markers, which are commonly used to enhance the precision of image alignment, and concluded that there was no significant difference in the quality of the co-registration with or without them. This implies that using these markers might be dispensable in SPECT/CT co-registration, simplifying the process and saving time. The authors also compared the performance of NMDose-coreg with that of OLINDA/EXM, which is considered the standard software for estimating the renal dose in patients undergoing ^177^Lu-DOTATATE therapy-for the target organ and for the surrounding tissues. They demonstrated that NMDose-coreg reduced the total processing time without compromising image quality after co-registration. These results are promising for applying NMDose-coreg in clinical practice, especially considering its practical implications in time-saving and process simplification. However, there were significant differences between the two methods, in terms of the doses calculated, underscoring the need for multicenter studies involving larger samples in order to understand the exact causes of those differences.

The Leitão et al.^(3)^ study also opens new perspectives for preprocessing methods that enable greater accuracy when establishing absorbed dose values, for the target organ and for other tissues. Artificial intelligence resources applied in structural segmentation or rigid image registration hold promise for optimizing absorbed dose planning for TRT. That innovation could facilitate the incorporation of internal dosimetry as a standard practice in personalized medicine. The list of therapies involving radioisotopes continues to expand, which makes maintaining high radiological protection standards for patients of paramount importance. This movement aligns with rigorous, optimized protocols and harmonizes with initiatives like the one established in European Council Directive 2013/59/Euratom^(7)^.

Although many challenges lie ahead, a thorough understanding of the workflow for implementing personalized internal dosimetry planning will contribute significantly to developing and refining planning models. Ideally, such models will be standardized, replicable, and traceable, which will enhance the safety and effectiveness of radioisotope therapies.
